# Variation in Prescription Opioid Dispensing across Neighborhoods of Diverse Socioeconomic Disadvantages in Victoria, Australia

**DOI:** 10.3390/ph11040116

**Published:** 2018-11-01

**Authors:** M Mofizul Islam, Dennis Wollersheim

**Affiliations:** 1 Department of Public Health, La Trobe University, Melbourne, Victoria 3086, Australia; 2 Health Information Management, School of Psychology and Public Health, La Trobe University, Melbourne, Victoria 3086, Australia; D.Wollersheim@latrobe.edu.au

**Keywords:** geographical variation, dispensing, prescription opioid, pain medicine, Victoria, Australia

## Abstract

The study examined the relationship between dispensing patterns of prescription opioids, neighborhood-disadvantage-index, and standardized doses dispensed. Three-year’s dispensing data drawn from 80 local government areas (LGAs) within Victoria, Australia’s second most populous state, was analyzed. Quantities dispensed in defined daily dose (DDD)/1000-people/day were computed for LGAs of low, moderate, high, and very high socio-economic disadvantage. LGAs with various levels of dispensing, and neighborhood disadvantage were identified and mapped. A multivariable regression model examined the effect of neighborhood level disadvantage and identified other factors that are associated with standardized doses dispensed. More women were dispensed opioids than men. Dispensing increased with increasing age. Most of the LGAs with relatively high dispensing were socioeconomically disadvantaged and located outside the major cities. Dispensing gradually increased from low disadvantage to very high disadvantage areas. Dispensing of standardized doses were consistently higher in rural areas than in urban areas. Neighborhood level disadvantage, age, sex, and urbanization were significant factors in the standardized doses dispensed. As inappropriate dispensing of opioids is a major public health problem, research should facilitate understanding of utilization in small areas to enable tailored public health programs. Nationwide and consistent introduction of real-time prescription drug-monitoring programs, and structural interventions to reduce the fundamental causes of socioeconomic disadvantage and isolation are recommended.

## 1. Introduction

Opioids act on the nervous system to relieve pain. However, opioids are addictive and may cause dependence, particularly among the long-term users [1,2]. Consequently, over recent years, recreational use, misuse, overdoses, and associated deaths from prescription opioids have increased alongside the medical use of these medicines [3,4,5]. For instance, in Australia during 2011–2015, more than 3000 people died from opioid-related overdose—a nearly twofold increase from the previous decade [6,7]. More recently, harms resulting from prescription opioid misuse exceeded the magnitude of problems from illicit drugs [8,9]. For instance, between 2011 and 2015, in Australia, there were more deaths (n = 2145) associated with oxycodone, morphine, codeine, fentanyl, tramadol and/or pethidine than heroin (n = 985) [9]. Due to these adverse health outcomes and their growing trends, and a widely appreciated connection with opioid dispensing [10,11], the misuse of prescription opioid has become a major public health concern in many countries. Relevant policies and interventions are mostly trying to reduce inappropriate dispensing and the utilization of these medicines.

Of note, there exists a considerable geospatial variation in opioid dispensing [12,13,14]. Relative socio-economic condition of the population in respective locations is likely to be the dominant factor determining geographical variability of prescription opioid use. As a result, studying the relationships between prescription patterns of opioid, quantity dispensed, and individuals who use opioid and their socio-economic conditions at relatively small geographies could facilitate better understanding of the problem. This relationship may help to implement local-level interventions [15]. However, literature around effect on neighborhood socioeconomic disadvantage on prescription opioid use is scarce and findings are inconsistent. For instance, Joynt et al. [16] found people living in a neighborhood of relatively low socioeconomic status were less likely to receive an opioid prescription. On the other hand, Ndlovu et al. [17] found that people living in a relatively high level of neighborhood disadvantage were more likely to receive strong analgesic (mostly opioid) therapy. This discrepancy warrants more research.

Research so far conducted in Australia on dispensing of prescription opioids mostly used national or state level data [18,19], with limited focus on small geographical level dispensing [20]. As a result, little is known about the relationship between prescription opioid dispensing and neighborhood level disadvantage. Therefore, we examined (i) variation and trends in prescription opioids dispensing in Victoria (Australia’s second most populous state) and (ii) relationship of quantity dispensed with neighborhood level disadvantage and rural-urban locality.

## 2. Results

**Figure 1:**
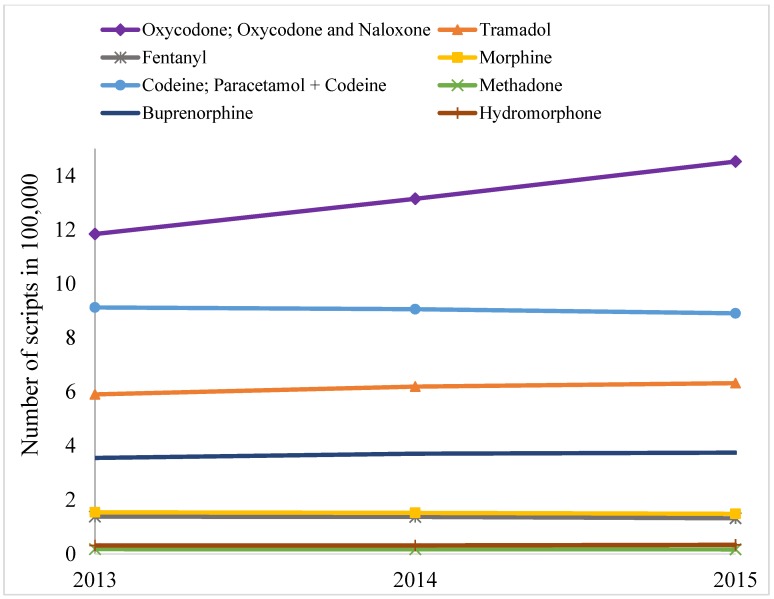
Annual trends in number of scripts during 2013–2015.

**Figure 2:**
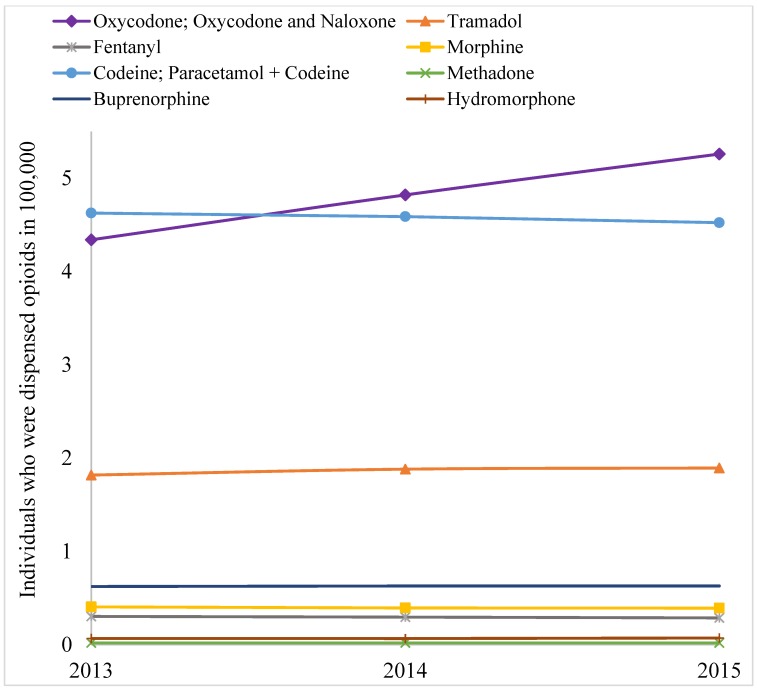
Items dispensed to individuals during the study period.

Over the study period (1 January 2013–31 December 2015) the number of opioid scripts remained mostly static for most items, except for oxycodone and derivatives, which increased substantially (Figure 1). Similarly, number of individuals to whom opioids were dispensed also increased for oxycodone and derivates (Figure 2), but quantities dispensed declined slightly in terms of DDD/1000 people/day (Table 1). Over the three years, a total of 10.68 million opioid prescriptions were written. In terms of DDD/1000 people/day, codeine and its derivatives dominate the quantity of individual items dispensed, across the years, followed by oxycodone (and derivatives) and tramadol (Table 1).

However, more prescriptions were written for oxycodone (and derivatives) than any other items, and there was a substantial increasing trend in prescription for oxycodone (and derivatives) between 2013 and 2015 (Figure 1). The number of individuals that were dispensed prescription opioid was the highest for oxycodone (and derivatives), followed by codeine and tramadol (Figure 2). The trend for number of individuals who were dispensed oxycodone (and derivatives) was significantly positive across the years. Overall, codeine, oxycodone, and tramadol were the main opioids dispensed in Victoria in three measures: number of prescriptions dispensed, individuals to whom opioids were dispensed, and quantities in DDD/1000 people/day.

Women were dispensed consistently higher quantities of opioids in terms of DDD/1000 people/day than men across the years and SEIFA categories (Table 2). Also, more women were dispensed opioids than men. On average in each year during the study period around 714,775 women received prescriptions as compared to 559,292 men. Consistently, the rate of prescription per user was higher for women than men.

There was an age trend in dispensing over the years, with consistently highest dispensing among people aged 65 years or older. Around half of the Victorians in this senior age-group were dispensed opioids in one or more occasions in each of three years. Among the 20–44 age-group around more than a quarter were dispensed opioids. There were slight increments across the years for all four age groups in terms of proportions of Victorians who were dispensed opioids, and the rates of increment were similar (details not shown).

There was an increasing trend in dispensing across the four layers of SEIFA—gradually increasing from very high SEIFA (i.e., low disadvantage) areas to low SEIFA (i.e., very high disadvantage) areas (Table 3). This trend was consistent across the years, and for men and women (Table 2).

Multivariable regressions were consistent to the univariate findings in that the women were likely to be dispensed higher doses of prescription opioid than men. There was a gradual increment in regression coefficient for age and it peaked for 65+ age group (Table 4). Dispensing of standardized doses decreased significantly in 2014 compared to 2013.

People living in high, moderate, or low SEIFA areas were likely to use more quantities of opioid (*p* < 0.01) as compared to people living in the very high SEIFA areas, and there appears to be a gradient in this relationship (Table 2). Across the years people living in rural areas were consistently dispensed higher standardized doses than those living in urban areas. There was 143% difference in dispensing of prescription opioids in standardized doses between urban and rural areas (B = 2.43, 95%CI 2.36–2.51) (Table 4).

**Figure 3:**
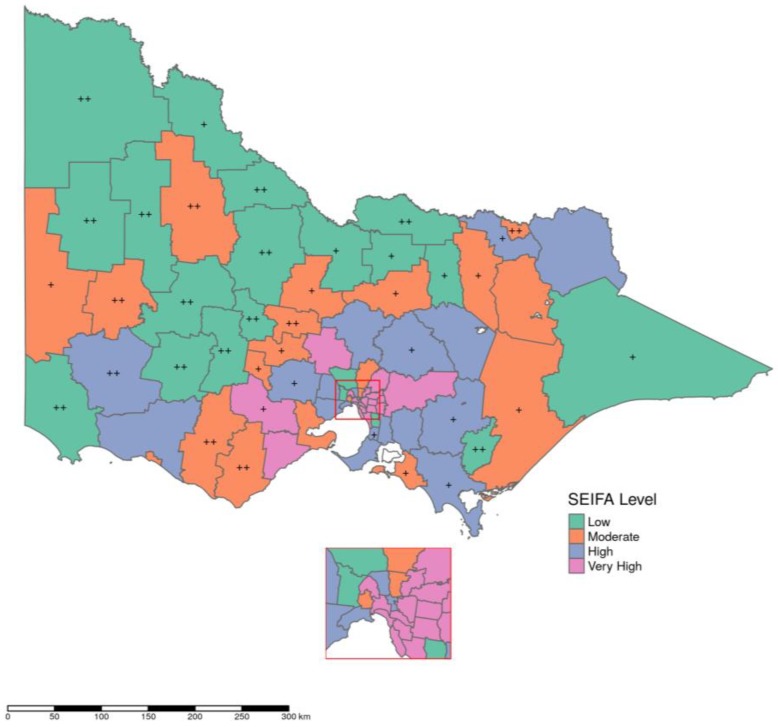
Dispensing of opioids in 2015 in local government areas (LGAs) of four types of neighborhood. LGAs with very high dispensing were marked with ++; high dispensing were marked with +; and, normal dispensing were marked with no sign.

**Figure 4:**
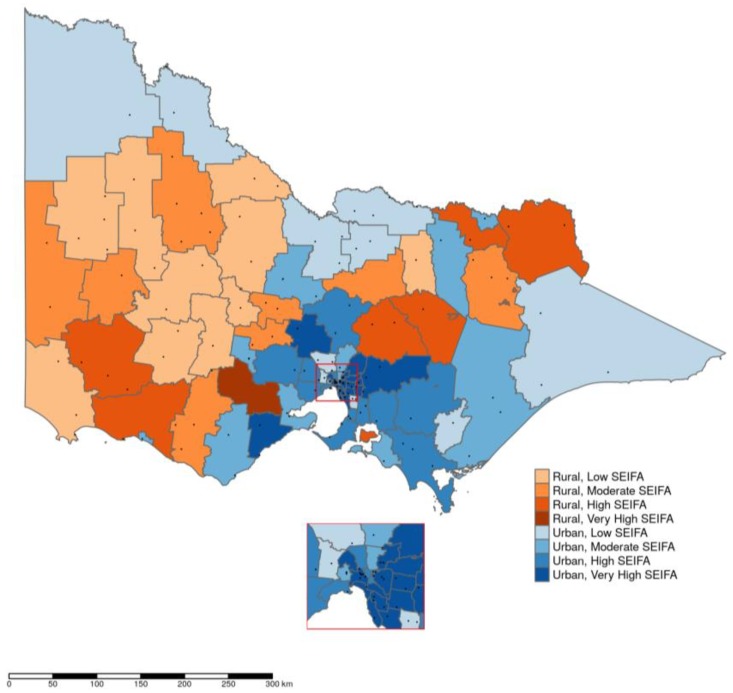
Dispensing of opioids in 2015 in LGAs of four SEIFA categories and rural and urban localities. Dots are pointing the location of hospitals and or clinics.

The average quantity of dispensing in terms of DDD/1000 people/day adjusted for age and sex remained mostly similar across three years of 2013–2015. Taking all 80 local government areas (LGAs) together, it was estimated that, in 2015, on an average 20 DDD/1000 people/day were dispensed per LGA with standard deviation ±15.27 DDD/1000 people/day. The differences between DDD/1000 people/day for individual LGAs and DDD/1000 people/day for state average were very high for 20 LGAs, high for 20 LGAs, and normal for the rest. Most of the LGAs with very high dispensing were in the group of lowest SEIFA scores (Figure 3) and outside major metropolitan cities (Figure 4). Across the LGAs, quantities dispensed in terms of DDD/1000 people/day over the years (2013–2015) had diverse trends in successive years, increasing in 13, decreasing in 35, and mixed in the remaining 32 LGAs (i.e., increasing in one year but decreasing in the other two years or vice versa). Among the LGAs with decreasing trend of dispensing, 20 had either high or very high SEIFA score and the remaining 15 had medium or least score. On the other hand, among LGAs with increasing trend of dispensing eight were of least or medium and five were of high or very high SEIFA score.

## 3. Discussion

The findings of this study suggest a considerable variation in dispensing of prescription opioids in Victoria. Overall the trend in number of prescriptions written for opioids remained almost flat for all items except oxycodone and derivates. A similar trend was observed in terms of the number of individuals who were dispensed opioids. However, dispensing in terms of DDD/1000 people/day slightly declined during the study period. Dispensing of total quantities and standardized doses were significantly higher in LGAs with relatively high level of disadvantage compared to LGAs with relatively low level of disadvantage. There was a significant increasing trend in dispensing of standardized doses across LGAs of four categories of neighborhood socioeconomic status. The findings showed that more women were dispensed an opioid than men. The proportion of Victorians who were dispensed an opioid increased in all age groups and across four categories of SEIFA over the study period. Among the individual medications, oxycodone prescriptions were rising faster than any other opioid formulation.

We found neighborhood level variation in dispensing, and a clear and significant trend in standardized doses dispensed across LGAs of four levels of SEIFA. This observation is similar to that found in another similar state—New South Wales [21] and therefore suggest that there is a relationship of prescription opioids use and area level socioeconomic disadvantage. This large difference is unlikely to be attributed solely to misuse or non-medical use. There is now growing body of evidence that suggest chronic pain is on the rise [22]. These observations, together, possibly suggest that the prevalence and or severity of pain and thereby levels of prescription opioids use are associated with the social determinants of health [23]. Also, people living in very high disadvantaged areas are more likely to have concession cards, enjoy lower copayments and hence have less barrier to opioid use when compared to people who live in lower disadvantaged areas [24].

Significantly more opioids were dispensed in terms of DDD/1000 people/day in LGAs that are mainly “rural” than in LGAs that are categorized mainly as “urban”. Higher level of dispensing in rural than urban areas could be a reflection of inadequate options of alternative pain treatment/management in rural or remote locations. This may also partly be attributed to the fact that the prescribing practices among the physicians are relatively relaxed in regional areas and this is somewhat influenced by a long-term relationship between the physicians and the patients and socio-cultural aspect of healthcare provision [25].

Our findings suggest sex and age were two important factors for overall quantity (in terms of DDD/1000 people/day) and standardized doses dispensed. This observation is consistent with the findings of previous studies [11,26,27]. There is a greater propensity among women to seek health care. Also, women live longer than men. Moreover, women are more likely to experience chronic pain and use pain medicine for longer periods and in higher doses than men [28]. Women who use opioids are more likely to become dependent more quickly and experience more cravings than men [29]. Furthermore, psychological distress among women were found to be a risk factor for the high-level use of prescription opioids. However, this association was not found significant among men [30]. The reason for substantial dispensing of opioids among the people of older age group could be explained by the fact that the prevalence of pain is relatively high among senior people.

There could be several reasons for the overall rising trend in number of scripts, and proportion of citizens who were dispensed opioids. The awareness of pain relief, prevalence, and the severity of illnesses causing chronic pain are increasing, which is mainly due to the ageing population [31]. There also appears to be a cultural shift—both among patients and health care providers—towards treating all types of pains with medicines [32]. Furthermore, prescribing opioids for patients is easy and fast, but having a comprehensive plan for pain management and implementing that plan is complex, time demanding, and rarely financially rewarded. Aggressive marketing efforts by the pharmaceutical industries [33] and increasing illicit use of prescription opioids may also have some effects on this rising trend in dispensing [34,35]. The increasing trend in script number was mostly attributed to increase on oxycodone (and derivatives), as shown in Figure 1 and Figure 2. However, total quantities in terms of DDD/1000 people/day declined slightly over the years. A closer examination revealed that the quantity of dispensing of oxycodone-naloxone combination tablet increased substantially, whereas dispensing of general oxycodone pills/capsules decreased to a certain extent. DDD quantity for oxycodone-naloxone combination tablet is much smaller than oxycodone pills/capsules. Thus, the increment by combination pill was offset by the declining dispensing of oxycodone pills/capsules, and the resulting quantities in terms of DDD/1000 people/day remained almost unchanged.

The combination of prolonged-release oxycodone with naloxone is marketed with an aim to getting dual effect—reduce pain and opioid-induced constipation. The addition of naloxone to oxycodone formulation aims to counteract the effect of oxycodone in the gastrointestinal tract over 12 h, as it antagonises opioid receptors resulting in increased gastric motility and overall reduction in opioid-induced constipation [36]. This combination drug is used to relieve moderate to severe persistent pain particularly when other medicines are found ineffective [37]. The rising dispensing of this combination item might be attributed to several factors, such as the growing number of ageing people and high prevalence of persistent pain patients, increasing trends in cancer incidence, and a declining trend in deaths that are attributable to cancer in Victoria [38], and new knowledge about improved safety and decreased adverse effect of oxycodone-naloxone. Also, a relatively low abuse potential of oxycodone-naloxone combination formulation is considered to be a favourable pharmacological feature and might be preferred by both prescribers and users [9].

Findings of this study aim to measure the relationship between dispensing and area level socioeconomic disadvantage and does not aim to suggest whether dispensing was appropriate. A relatively high dispensing does not necessarily indicate an inappropriate utilization. However, undue use of this medicine and harms from that use are a public health issue. SEIFA indicates an overall area-level disadvantage status and not all people living in an area have the same level of disadvantage [39]. As a result, findings of this study warrant small area-based research to identify the factors leading to high dispensing and the loading of component variables that determine the level of utilization of opioids. Also, rigorous qualitative studies are needed for an in-depth understanding of the extent and circumstances in which opioids are being used in instances other than for which they are prescribed. To improve policy and practice around appropriate dispensing of opioids, multidisciplinary research is required.

Ensuring appropriate use of opioids in population level is a difficult task and it demands a balance so that the pain patients are not deprived of opioids. Both short and long-term programs should be taken to achieve this balance. In the short-term, the introduction of a real-time drug-monitoring program and access to that database by both doctors and pharmacists at point-of-care may reduce excessive utilization of opioids [40,41,42]. The state-based monitoring programs using different software may hinder the interoperability across the states [43]. As a result, a nationwide initiative of this monitoring program using a same software is likely to be more effective. Long-term programs should aim at structural interventions to eliminate the root causes, as the high utilization of opioids is likely to be influenced by economic and social upheaval, concentrated disadvantage, and isolation [23].

Our study has several strengths and limitations. We analyzed almost the complete dataset, rather than a sample. We also adjusted our regression model for cancer cases across the LGAs. However, only three-year’s data was available. Also, opioids that were dispensed in hospitals and through private prescriptions were not captured in this dataset. The DDD is a technical unit and does not always correspond to the recommended daily dose for every person. The dataset we collected had only a limited set of variables. The analysis could have offered more information had there been more demographic variables such as race, ethnicity, educational, and marital status, etc. Ecological fallacy is another limitation, as not everybody living an area are similar [44]. Also, SEIFA offers little information about the particular types of disadvantage at the individual or area-level. The use of any medicines is usually associated with a range of factors; age, sex, and neighborhood level socioeconomic conditions are some of them, and they do not offer the complete picture. A range of other factors may influence the level of dispensing, including the availability of local support structures in neighborhoods, such as local community outreach groups, churches, and non-profit organizations, and can counteract dispensing and misuse of opioids. We could not asses that relationship. Furthermore, the dispensing across three-year period may also have been influenced by structural changes that took place around medicare, PBS, and or institutional changes for healthcare providers. Future research should consider more detailed information.

## 4. Conclusions

There were geographical and temporal variations in prescription opioid dispensing in Victoria during the study period. Most of the LGAs with relatively high dispensing were socioeconomically deprived and located outside the major cities. There was an increasing trend in dispensing across four categories of SEIFA—gradually increasing from low disadvantaged areas to very high disadvantaged areas. Neighborhood level disadvantage, age, and sex are three significant factors in standardized doses dispensed. Very high levels of dispensing in terms of DDD/1000 people/day were recorded in a number of LGAs, which warrant further investigation to identify the factors, more precisely. As continuing use of opioids has considerable side effects and excessive use is a key public health concern, further research is warranted to understand the primary reasons for such usages in small areas so that tailored interventions could be implemented. Both short- and long-term programs are recommended. Long-term programs should aim at structural interventions to reduce the fundamental causes of socioeconomic disadvantage and isolation.

## 5. Materials and Methods

### 5.1. Dispensing of Prescription Opioids in Australia

In Australia, more than 80% of all community prescriptions (i.e., non-public hospital) are dispensed under one of two subsidized schemes—the Pharmaceutical Benefits Scheme (PBS) and the Repatriation Pharmaceutical Benefits Scheme (RPBS) [45]. In PBS, patients pay a co-payment (currently $6.40 for concession card holders and up to $39.50 for general patients) and Government subsidizes the remaining cost. There is also a safety net threshold on PBS items. When threshold is reached, general patients are entitled to PBS medications at the concession price; and concessional patients are entitled to PBS medications at no cost—for the rest of that calendar year. In RPBS, which was introduced for the military veterans, patients pay the same copayments as concessional patients. Some medicines are not listed in PBS/RPBS. For these medicines, the patient pays full price, and these are not counted towards the safety net threshold [45] and are not included in this dataset. A number of PBS items are priced below general patient co-payment; and thus, patients pay the full payment, which is counted towards the safety net threshold and these prescriptions are included in this dataset.

### 5.2. Dataset

Opioids dispensing data was provided by the statistics branch of the Commonwealth/Federal Department of Human Services. This study used three-year (2013–2015) dispensing data of prescription opioids in the second most populated state, Victoria. The dataset was extracted based on the date of supply. The dataset contained the total quantity, number of prescriptions and number of persons dispensed for each combination of the variables: sex, age group (0–19, 20–44, 45–64 and, 65+ years), opioid name, and LGA. A sample of data structure is available in the Supplementary Table S1. Demographic data for population of individual LGAs, and index of relative socio-economic disadvantage score of Socio-Economic Indexes for Areas (SEIFA), which ranks areas according to relative socio-economic disadvantage were obtained from the Australian Bureau of Statistics. SEIFA data are derived from information collected in five-yearly national census and are based on a range of variables, including education, occupation, employment, income, families, and housing, etc. SEIFA is an area-level relative measure, an average—a summary measure of the people and households within an area and may not necessarily represent the individual status of a person or household within the area. Higher SEIFA scores indicate an overall lower level of disadvantage and lower scores indicate a higher level of disadvantage [39]. It is a marker of relative socio-economic disadvantage in terms of people's access to material and social resources, and their ability to participate in society. Cancer prevalence data was collected from the Victorian Cancer Registry Office.

### 5.3. Data Analysis

We used defined daily dose (DDD) unit, as introduced by the WHO Collaborating Centre, to quantify drugs dispensed across different types of opioids [46]. DDD corresponds to the estimated defined daily dose of a drug when used for its main indication in adults. Using this DDD unit, we quantified all items to defined daily dose per 1000 people per day (indicated as DDD/1000 people/day), and thus each cell of quantity (please see the Supplementary Table S1) was converted to DDD/1000 people/day—which is, in fact, a standardized dose of individual item for men or women of each age-group in an LGA in a given year. The quantities in DDD/1000 people/day were also computed to get an estimate of aggregate dispensing for individual opioid types, LGAs, age-groups, sex and four types of SEIFA. The advantage of DDD/1000 people/day is that this unit simplifies comparisons of medicine use across populations and medicine types.

The proportion of population who were dispensed opioids was computed for four age groups (0–19, 20–44, 45–64, 65+) and for each of the study years between 2013 and 2015. We used Chi-squared test of trend for proportions [47] to determine significant differences over time. LGA urbanization level—as rural or urban—was determined by the Australian Classification of Local Government categorized in 2013 [48].

Using the direct standardization approach [49] the quantities dispensing in terms of DDD/1000 people/day in individual LGAs were adjusted for the population structure of overall Victoria, stratified across age and sex. The differences between “average quantities dispensed in DDD/1000 people/day for overall Victoria” and “age and sex adjusted DDD/1000 people/day quantities dispensed for individual LGAs” were computed to categorize LGAs with relatively high or low dispensing rates. LGAs were identified as ‘normal’ if dispensing was less than or equal to the state average; ‘high’ if dispensing was between state average and the third quartile; and, ‘very high’ for anything more than that. LGAs of these three categories along with their respective SEIFA level were then mapped using R software version 3.4.4 [50] and the tidyverse and tmap packages [51,52] to generate the maps.

A multivariable regression model was developed to examine the effect of neighborhood level disadvantage and to identify other factors associated with the quantity of standardized doses of individual item dispensed for men or women of each age-group in an LGA in a given year. As the variable ‘standardized dose’ was positively skewed we used a logarithmic transformation. To make the regression coefficients (β) interpretable they were exponentially transformed and reported as value ‘B’. The interpretation of B is that a one unit increase of the independent variable would result in a (B-1)*100 percentage change in the standardized doses dispensed per person [53]. The SEIFA scores were divided into quartiles for modelling purposes, as a linear relationship does not exist.

STATA SE 14 (StataCorp LP), Microsoft Office Excel 2013 and R were used for analyses.

### 5.4. Ethics Approval and Consent to Participate

The study received approval from La Trobe University’s Ethics Committee (reference number: S17-218).

## Figures and Tables

**Table 1:** Quantity of major opioids dispensed under Pharmaceutical Benefits Scheme (PBS), Repatriation Pharmaceutical Benefits Scheme (RPBS), and under-copayment scheme (in DDD/1000 people/day).

Generic Drugs	2013	2014	2015
Tramadol	2.74	2.79	2.76
Codeine and derivatives ^§^	6.16	6.04	5.76
Oxycodone and derivatives ^¶^	3.10	3.12	3.18
Fentanyl	1.02	0.96	0.89
Morphine (& derivatives)	1.03	0.93	0.85
Buprenorphine	0.68	0.70	0.70
Hydromorphone	0.32	0.30	0.33
Methadone	0.30	0.28	0.27
Tapentadol	-	0.05	0.22

Note. ^§^ Codeine and paracetamol combination; ^¶^ Oxycodone and naloxone combination.

**Table 2:** Opioids dispensed over the years, across SEIFA level for men and women.

Year	Quantity Dispensed in Terms of DDD/1000 People/Day							
	Low SEIFA		Moderate SEIFA		High SEIFA		Very High SEIFA	
	Men	Women	Men	Women	Men	Women	Men	Women
2013	18.92	22.25	16.95	20.69	14.12	17.76	8.71	11.96
2014	18.56	22.34	16.69	20.76	13.80	17.84	8.52	11.66
2015	18.43	22.13	16.46	20.86	13.57	17.51	8.24	11.45

**Table 3:** Opioid dispensing during 2013–2015 across four levels of Socio-Economic Indexes for Areas (SEIFA).

Year	Variable	SEIFA Index			
		Low	Moderate	High	Very High
2013	Population (in 100,000)	9.50	11.22	15.06	21.56
	Number of individuals who were dispensed prescription opioids (in 100,000)	2.36	2.66	3.31	3.75
	Proportion of population that were dispensed prescription opioids (in %)	24.84	23.71	21.98	17.39
	Amount of prescription opioids used (in DDD/1000 people/day)	20.58	18.84	15.95	10.36
2014	Population (in 100,000)	9.61	11.43	15.46	21.88
	Number of individuals who were dispensed prescription opioids (in 100,000)	2.49	2.78	3.50	3.88
	Proportion of population that were dispensed prescription opioids (in %)	25.91	24.32	22.64	17.73
	Amount of prescription opioids used (in DDD/1000 people/day)	20.45	18.74	15.84	10.12
2015	Population (in 100,000)	9.70	11.63	15.86	22.18
	Number of individuals who were dispensed prescription opioids (in 100,000)	2.57	2.90	3.67	4.00
	Proportion of population that were dispensed prescription opioids (in %)	26.49	24.94	23.14	18.03
	Amount of prescription opioids used (in DDD/1000 people/day)	20.28	18.68	15.56	9.87

Note. Trends for proportion of person used prescription opioids across years for all four SEIFA levels were significant and positive.

**Table 4:** Multivariable model reflecting factors associated with quantity of standardized doses dispensed.

Variables	B *	*P*	95% Confidence Interval
**SEIFA index level**			
Very high	1.00	-	-
High	1.21	<0.01	1.17–1.24
Moderate	1.48	<0.01	1.44–1.53
Low	1.60	<0.01	1.55–1.65
Sex			
Men	1.00	-	-
Women	1.14	<0.01	1.12–1.17
**Age group (years)**			
0–19	1.00	-	-
20–44	4.81	<0.01	4.60–5.04
45–64	11.05	<0.01	10.55–11.57
65+	18.69	<0.01	17.79–19.64
**Urbanization**			
Urban	1.00	-	-
Rural	2.43	<0.01	2.36–2.51
**Year**			
2013	1.00	-	-
2014	0.96	<0.01	0.94–0.99
2015	0.98	0.28	0.96–1.01
Cancer prevalence	1.00	<0.01	1.00–1.00
Constant	1.02	0.38	0.97–1.07

Note. * Exponentiated values.

## Supplementary Materials

The following are available online at http://www.mdpi.com/1424-8247/11/4/116/s1, Table S1: Sample of drug dispensing dataset received from the department of health.
