# Supplementary material for: Variation in Prescription Opioid Dispensing across Neighborhoods of Diverse Socioeconomic Disadvantages in Victoria, Australia
Source: Pharmaceuticals (Basel). 2018 Nov 1;11(4):116. doi: 10.3390/ph11040116 (PMC6315505; doi:10.3390/ph11040116)
Supplement: Supplementary file 1 [file pharmaceuticals-11-00116-s001.docx]

**Supplementary Table S1:** Sample of drug dispensing dataset received from the department of health.

| **State** | **Supply year** | **LGA code** | **ATC** | **Item name** | **Item code** | **Item form** | **Scheme** | **Sex** | **Age** | **No. scripts** | **Supplied quantity** | **No.**  **patients** |
| --- | --- | --- | --- | --- | --- | --- | --- | --- | --- | --- | --- | --- |
| VIC | 2013 | 21450 | N02AA01 | MORPHINE (PLC)(5392T) | 01653B | 10MG TAB MOD REL SULF, 28 TABS -DOCT | UNDERCO | F | 0-19 | 1 | 28 | 1 |
| VIC | 2013 | 21450 | N02AA05 | OXYCODONE | 08386J | 20MG TAB MOD REL HCL, 28 TABS -DOCT | PBS | F | 0-19 | 1 | 28 | 1 |
| VIC | 2013 | 21890 | N02AA01 | MORPHINE (PLC)(5392T) | 05392T | MORPHINE SULFATE 200MG TABLET: MODIFIED RELEASE, 28 TABLETS | PBS | F | 65+ | 2 | 56 | 1 |
| VIC | 2013 | 22170 | N02AA01 | MORPHINE (PLC)(5392T) | 05392T | MORPHINE SULFATE 200MG TABLET: MODIFIED RELEASE, 28 TABLETS | PBS | M | 65+ | 5 | 560 | 2 |
| VIC | 2013 | 23270 | N02AA01 | MORPHINE (PLC)(5392T) | 05392T | MORPHINE SULFATE 200MG TABLET: MODIFIED RELEASE, 28 TABLETS | PBS | F | 65+ | 2 | 56 | 1 |
| VIC | 2014 | 23670 | N02AA01 | MORPHINE (PLC)(5392T) | 05392T | MORPHINE SULFATE 200MG TABLET: MODIFIED RELEASE, 28 TABLETS | PBS | F | 45-64 | 5 | 140 | 1 |
| VIC | 2014 | 23670 | N02AA01 | MORPHINE (PLC)(5392T) | 05392T | MORPHINE SULFATE 200MG TABLET: MODIFIED RELEASE, 28 TABLETS | PBS | F | 45-64 | 2 | 280 | 5 |
| VIC | 2014 | 24210 | N02AA01 | MORPHINE (PLC)(5392T) | 05392T | MORPHINE SULFATE 200MG TABLET: MODIFIED RELEASE, 28 TABLETS | PBS | F | 65+ | 1 | 56 | 2 |
| VIC | 2014 | 24650 | N02AA01 | MORPHINE (PLC)(5392T) | 05392T | MORPHINE SULFATE 200MG TABLET: MODIFIED RELEASE, 28 TABLETS | PBS | M | 45-64 | 1 | 56 | 2 |
| VIC | 2015 | 24900 | N02AA01 | MORPHINE (PLC)(5392T) | 05392T | MORPHINE SULFATE 200MG TABLET: MODIFIED RELEASE, 28 TABLETS | PBS | M | 45-64 | 1 | 28 | 1 |
| VIC | 2015 | 27260 | N02AA01 | MORPHINE (PLC)(5392T) | 05392T | MORPHINE SULFATE 200MG TABLET: MODIFIED RELEASE, 28 TABLETS | PBS | M | 65+ | 1 | 28 | 1 |
